# Non-invasive prenatal diagnosis (NIPD): current and emerging technologies

**DOI:** 10.20517/evcna.2022.44

**Published:** 2023-02-22

**Authors:** Britt Hanson, Ben Paternoster, Nikita Povarnitsyn, Elizabeth Scotchman, Lyn Chitty, Natalie Chandler

**Affiliations:** ^1^North Thames Genomic Laboratory Hub, Great Ormond Street NHS Foundation Trust, London WC1N 3BH, UK.; ^2^Genetic and Genomic Medicine, UCL Great Ormond Street Institute of Child Health, London WC1N 1EH, UK.

**Keywords:** Cell-free DNA, non-invasive prenatal diagnosis, monogenic conditions

## Abstract

Prenatal testing is important for the early detection and diagnosis of rare genetic conditions with life-changing implications for the patient and their family. Gaining access to the fetal genotype can be achieved using gold-standard invasive sampling methods, such as amniocentesis and chorionic villus sampling, but these carry a small risk of miscarriage. Non-invasive prenatal diagnosis (NIPD) for select rare monogenic conditions has been in clinical service in England since 2012 and has revolutionised the field of prenatal diagnostics by reducing the number of women undergoing invasive sampling procedures. Fetal-derived genomic material is present in a highly fragmented form amongst the maternal cell-free DNA (cfDNA) in circulation, with sequence coverage across the entire fetal genome. Cell-free fetal DNA (cffDNA) is the foundation for NIPD, and several technologies have been clinically implemented for the detection of paternally inherited and *de novo *pathogenic variants. Conversely, a low abundance of cffDNA within a high background of maternal cfDNA makes assigning maternally inherited variants to the fetal fraction a significantly more challenging task. Research is ongoing to expand available tests for maternal inheritance to include a broader range of monogenic conditions, as well as to uncover novel diagnostic avenues. This review covers the scope of technologies currently clinically available for NIPD of monogenic conditions and those still in the research pipeline towards implementation in the future.

## INTRODUCTION

### Cell-free DNA

During pregnancy, maternal blood plasma contains cell-free DNA (cfDNA) derived from organs and tissues throughout the maternal body, as well as the developing fetus^[1]^. Cell-free fetal DNA (cffDNA) primarily originates from placental trophoblastic cells^[2,3]^, is detectable in maternal circulation from ~4 weeks gestation, and is representative of the entire fetal genome^[4]^. cffDNA is shed into the bloodstream in a highly fragmented form^[5]^ and is specific to the ongoing pregnancy, being cleared rapidly following removal of the placenta^[6]^.

The ability to ascertain genetic information directly from cfDNA via non-invasive sampling methods has revolutionised the fields of oncology, transplant monitoring, and prenatal screening. Knowledge about affected pregnancies from the earliest timepoint provides valuable time for decision making regarding pregnancy management as well as therapeutic or surgical intervention *in utero*, or at the earliest possible postnatal timepoint. While there is an ongoing debate as to the relative risk of miscarriage associated with invasive sampling methods, such as amniocentesis and chorionic villus sampling (CVS)^[7,8]^, the success of the procedure is dependent on the skill of the healthcare professional, and surveys indicate that patients prefer non-invasive sampling via venous blood draw, where available^[9,10]^. Non-invasive acquisition of cfDNA can be carried out rapidly at low cost, and offers the potential for safe repeat sampling, if required.

The quantity of cffDNA amongst the high background of maternal cfDNA at any given timepoint during pregnancy is referred to as the fetal fraction (FF). It is generally accepted that FF increases with gestational age; however, there is still limited consensus as to the contribution of additional factors towards FF variability^[11]^. According to a large cohort study of 1,949 low- and high-risk singleton pregnancies, FF reportedly increases at a rate of 2.6% per week between gestation weeks 8-10, 0.2% per week between weeks 10-20, and 0.7% per week up to delivery^[12]^. On average, the FF is ~10% at the approximate timepoint from which invasive prenatal testing can typically be carried out (i.e., 11-13 weeks)^[12]^; however, this value can range from < 4% to > 30%^[13,14]^. While the use of long-read sequencing has been explored extensively for diagnostics in the postnatal setting, its application in the context of NIPD is somewhat lacking. Previously, the average length of cfDNA was reported to be ~143 bp for cell-free fetal DNA and ~166 bp for maternal cfDNA^[4]^. However, this observation has been confounded by the application of short-read sequencing technologies to cfDNA, which have a maximum size limitation of ~300 bp. Using Pacific Biosciences (PacBio, Menlo Park, CA, USA) single molecule real-time sequencing (SMRT) sequencing technology, it has been shown that cfDNA can exist as significantly longer fragments of up to ~23 kb^[15]^. The same study revealed that the proportion of cfDNA fragments over 500 bp increases with gestational age, accounting for 15.5%, 19.0%, and 32.3% of the total cfDNA during the first, second, and third trimesters of pregnancy, respectively^[15]^.

### Non-invasive prenatal diagnosis for monogenic conditions

In 2007, Lo *et al*. reported detection of Trisomy 21 in a fetus with Down syndrome via the direct analysis of plasma obtained from a maternal peripheral blood sample^[16]^. Since 2008, non-invasive prenatal testing (NIPT) for aneuploidy has been rapidly incorporated into routine clinical service, primarily for the screening of common aneuploidies, which include Patau syndrome (T13), Edwards syndrome (T18), and Down syndrome (T21)^[17]^. NIPT is available as a first-line or contingent test for intermediate- to high-risk pregnancies in over 60 countries worldwide and has significantly reduced the overall number of women undergoing invasive sampling procedures^[18]^. Even so, an invasive follow-up test is required for confirmation of a positive NIPT result owing to several potential confounding factors, such as confined placental mosaicism (CPM), identification of maternal chromosomal anomalies, circulating tumour DNA derived from maternal tumours and/or neoplasms, and cffDNA released from a vanishing twin^[19,20]^. On the contrary, when considering monogenic conditions in high-risk pregnancies where there is a known family history or an abnormal ultrasound finding (suggestive of a monogenic condition), non-invasive prenatal diagnosis (NIPD) of monogenic conditions does not require an invasive follow-up test. This is because CPM, while extremely rare^[21]^, is unlikely to be an issue, and the maternal genome is taken into account as this is analysed in parallel with the cfDNA.

NIPD has been available in the clinical setting since 2011 for fetal sex determination^[22,23]^ and diagnosis of fetal rhesus D blood group (*RHD*) status in RhD-negative women to prevent haemolytic disease of the fetus and newborn (HDFN)^[24-26]^. Non-invasive fetal ABO blood group prediction has also recently been clinically implemented as a means to reduce instances of HDFN resulting from other causal blood group markers^[27]^. Research into NIPD for monogenic conditions has been ongoing since 2000, with the first publications describing potential clinical utility for achondroplasia^[28]^ and myotonic dystrophy^[29]^. NIPD has now been implemented in clinical service in England for several monogenic conditions, including *FGFR2*- and *FGFR3*-related skeletal conditions^[30-33]^, *DMD*-related Duchenne and Becker muscular dystrophies^[34,35]^, cystic fibrosis^[36]^, and spinal muscular atrophy^[35,37]^. It is also available for families with rare monogenic conditions, but this has largely relied on bespoke test development for at-risk pregnancies owing to the rarity of individual cases and extensive mutational heterogeneity, which is both labour-intensive and costly to healthcare systems. Commercially available NIPT tests to screen low-risk pregnancies have recently also become available^[38,39]^, although the scope of possible diagnoses with these remains limited, and an invasive follow-up test is still required for confirmation of a positive result. Clinical implementation in accredited health service settings so far is only available for families at known increased risk because of a family history, or because ultrasound findings suggest a specific monogenic condition. In this review, we will largely focus on the technologies used for NIPD in these pregnancies at known increased risk.

It is generally accepted that NIPD can be performed at earlier timepoints than invasive sampling methods, which could provide valuable additional time for pregnancy management and decision making^[2,3]^. This, however, is dependent on the molecular diagnostic assay being used as well as the type of variant being detected. For instance, fetal sex determination can be carried out reliably from as early as 7 weeks gestation^[40]^, while the minimum gestation period for diagnosis of monogenic conditions is generally 8-9 weeks^[35]^.

At present, there is no all-encompassing technique allowing for accurate and sensitive detection of pathogenic variants in cfDNA. This is owing to the range of different genetic aberrations (from single nucleotide variants (SNVs) to larger copy number variants (CNVs), repeat expansions, and major chromosomal rearrangements) as well as the differences in the abundance of the pathogenic variant in the cfDNA dependent on their inheritance. Another important challenge faced by NIPD test development is the low-level of fetal-derived cfDNA that coexists amongst a high abundance of maternal cfDNA in circulation. Nonetheless, major strides have been made over the past two decades towards the development of several powerful molecular diagnostic technologies for NIPD of rare monogenic conditions to address the challenge of multiple modes of genetic inheritance, as well as mutational heterogeneity. This review will delve into greater detail on such technologies that are currently clinically available, as well as those being developed towards future clinical implementation.

## TECHNIQUES USED TO DETECT PATERNALLY INHERITED AND *DE NOVO* VARIANTS

**Figure 1:**
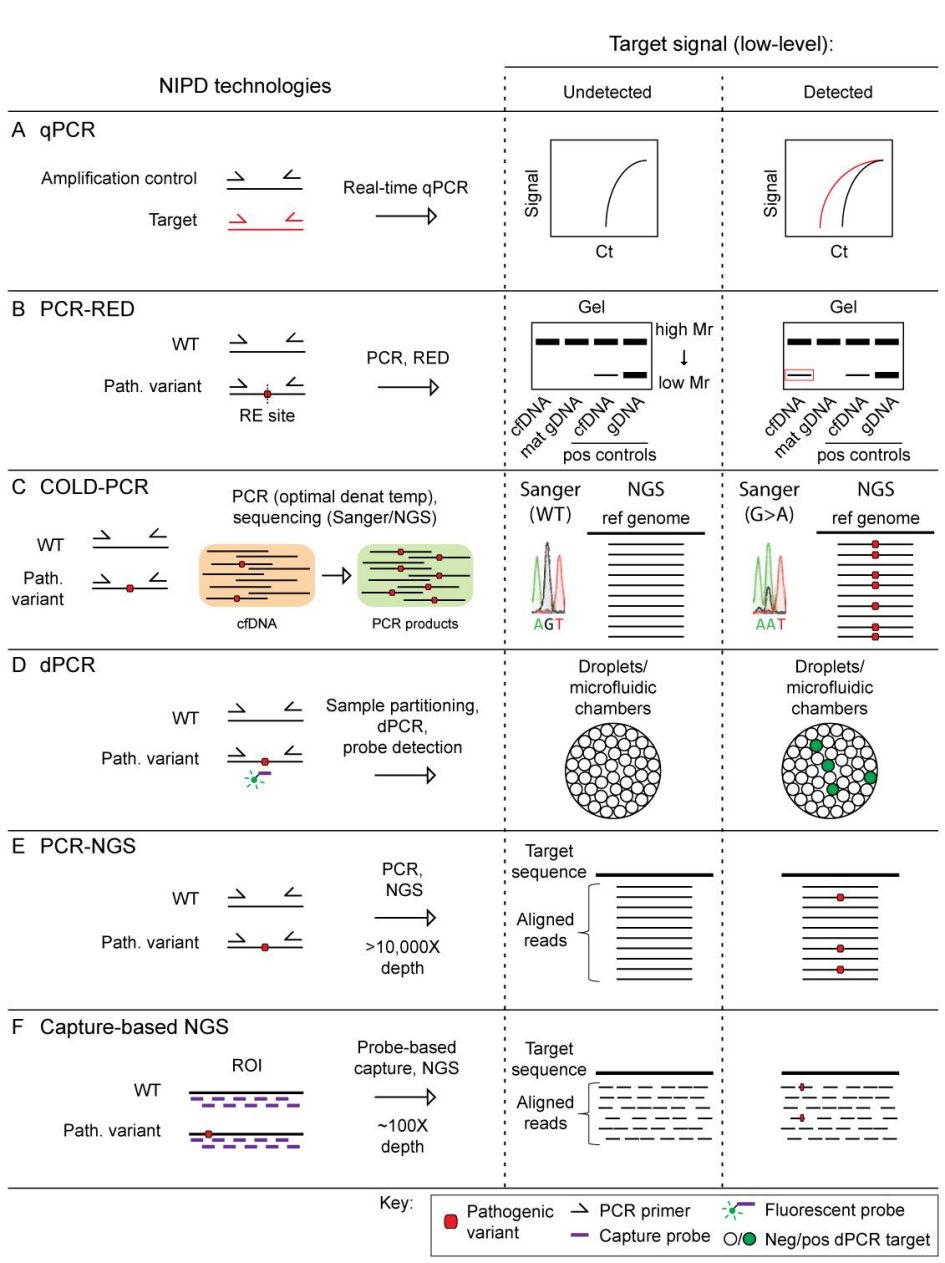
NIPD technologies for the detection of paternally inherited or *de novo* variants. Technologies developed for the detection of low-level paternally inherited or *de novo* variants. A: Real-time-quantitative polymerase chain reaction (RT-qPCR) determines the presence of a particular genomic target sequence above a PCR cycle threshold (Ct) set using a negative control. B: PCR followed by restriction enzyme digestion (PCR-RED) is applicable where the variant of interest creates a novel restriction enzyme (RE) recognition site otherwise absent in the wildtype (WT) target sequence. A typical negative control is maternal gDNA, while cfDNA and gDNA positive controls can also be run alongside. The digestion products are visualised by gel electrophoresis, where the presence of a lower molecular weight (Mr) band is indicative of a positive result (indicated by the red box in the diagram). C: Co-amplification at lower denaturation temperature-PCR (COLD-PCR) is applicable for targets where the variant allele creates a lower denaturation temperature than the WT allele, and can therefore be preferentially amplified using a modified PCR protocol (i.e., with a lower denaturation temperature during cycling). The variant-enriched product is detectable via Sanger sequencing or next-generation sequencing (NGS). D: Digital PCR (dPCR) is carried out using microfluidic devices or oil-water-emulsion technology (ddPCR) for compartmentalisation of individual genomic target sequences within nanolitre-sized chambers or droplets, respectively. Fluorescently labelled probes are designed with sequence homology to the target of interest such that recognition and binding generates a signal indicative of the presence of the target within the cfDNA sample. This signal can be quantified by counting the number of positive reaction units relative to the total number of reaction units containing a DNA molecule. E: PCR followed by next-generation sequencing (PCR-NGS) involves the use of targeted PCR for the enrichment of a short target region of interest (< 10 kb), followed by short-read sequencing at > 10,000X read depth (~150 bp per read). F: Capture-based NGS involves the use of targeted probes for the enrichment of a desired genomic locus (up to several Mb), followed by short-read sequencing to ~100X read depth for accurate variant detection without PCR-related artefacts.

Molecular diagnostic approaches towards detection of *de novo* and paternally inherited variants within the cfDNA of pregnant women are relatively straightforward as these genetic variants are confined to the FF. Such approaches can be applied to paternally inherited autosomal dominant conditions^[31,32]^, as well as for paternal exclusion testing in recessive conditions where the parents carry different mutations^[41,42] ^[Figure 1]. The approaches can also be applied in cases where the fetus is at germline mosaicism risk of autosomal dominant or X-linked conditions, or for screening for *de novo* mutations in fetuses with anomalies detected by imaging indicative of some single gene conditions (e.g., *FGFR2*/*FGFR3*).

### Real time-quantitative PCR

The first generation of tests to become clinically available for NIPD were based on targeted amplification polymerase chain reaction (PCR) technologies. Fetal sex determination was introduced into the clinic in 2011 through the use of real-time quantitative PCR (RT-qPCR) specific to Y chromosome genetic elements^[40] ^[Figure 1A]. This is an extremely valuable test in the context of X-linked or sex-limited conditions to restrict further testing to male fetuses or, in suspected cases of congenital adrenal hyperplasia (CAH), potentially providing dexamethasone treatment only to mothers with female fetuses^[43,44]^. qPCR is currently the leading method being employed for the clinical prediction of fetal *RHD* status^[24,25]^. The RT-qPCR workflow is laborious owing to the requirement for a standard curve and a separate assay for FF quantification, and thus some laboratories have introduced a digital PCR (dPCR) approach for fetal sex determination^[45]^ and paternal exclusion testing^[42]^ as this does not require a standard curve.

### PCR-restriction enzyme digestion

PCR followed by restriction enzyme digestion (PCR-RED) and gel electrophoresis was one of the first tests to enable NIPD of monogenic conditions arising from paternally inherited and *de novo* pathogenic variants [Figure 1B]. This technique was developed for common *FGFR3* pathogenic variants and was introduced into accredited clinical service in 2012^[30,31]^. PCR-RED involves PCR amplification of the target genetic locus and relies on differential restriction enzyme recognition patterns depending on whether the wildtype (WT) or pathogenic allele is present, providing a gel-based readout of expected digestion products. While PCR-RED is a technically straightforward and cost-effective methodology, disadvantages of this approach include subjectivity in user interpretation of the results, a limited scope and size range of pathogenic variants that can be assessed, and a high inconclusive rate of 8%^[31]^. It is also not possible to analyse more than one variant at a time. As such, PCR-RED has been superseded in clinical service by targeted PCR followed by next-generation sequencing (PCR-NGS) so as to broaden the scope of genes and variants that can be assessed, and to improve the accuracy and sensitivity of the assay^[32]^. This method is discussed later.

### Co-amplification at lower denaturation temperature-PCR

Co-amplification at lower denaturation temperature-PCR (COLD-PCR) followed by Sanger sequencing or NGS is a method that takes advantage of PCR amplification bias to preferentially amplify and detect minority alleles based on differences in denaturation temperature of the reference and alternate DNA sequences [Figure 1C]. Galbiati *et al*. assessed the use of COLD-PCR followed by Sanger sequencing for NIPD of several common variants (down to a level of < 1%) causative of β-thalassaemia^[46]^ and cystic fibrosis^[47]^, as well as a family-specific 18 bp deletion in *TWIST1*, causative of craniosynostosis^[48]^. This approach was validated against highly sensitive microarray assays^[47]^ and has also been applied in the research setting for NIPD of paternally inherited mutations causative of β-thalassaemia (*HBB*)^[49,50]^, and to detect feto-maternal platelet incompatibility^[51]^. This approach was validated against highly sensitive microarray assays^[47]^ and has also been applied in the research setting for NIPD of paternally inherited mutations causative of β-thalassaemia (*HBB* gene)^[49,50]^, and to detect feto-maternal platelet incompatibility.

### Digital PCR

dPCR technology offers enormous potential for novel diagnostic tools in the field of NIPD for monogenic conditions. dPCR enables binary end-point interrogation of the presence or absence of a target sequence of interest within individually contained reaction chambers^[52-54] ^[Figure 1D]. dPCR offers increased sensitivity over conventional RT-qPCR methods as sample dilution and compartmentalisation within discrete reaction units eliminates interference commonly caused by PCR inhibitors, and circumvents template-specific PCR biases (such as strand switching, and preferential amplification based on target size or variant-specific nucleic acid sequence differences)^[54-56]^. Furthermore, dPCR does not require standards or normalisation to achieve accurate absolute copy number quantification. The two leading dPCR systems that have been applied to NIPD development to date make use of microfluidic chambers^[52,53]^ or oil-water-emulsion technology resulting in droplet formation^[57]^ to achieve sample partitioning into tens of thousands of nanolitre-sized reaction units. While a major disadvantage of dPCR compared to qPCR is the increased cost, newer generations of dPCR systems have up to 6-colour detection capabilities, substantially increasing the multiplexing capacity and reducing the individual sample cost per experiment.

In relation to paternally inherited or *de novo* genetic variants, dPCR has been used to improve the sensitivity of fetal sex determination assays, allowing for application at earlier gestational timepoints (or generally for samples with lower FFs)^[45]^. Proof-of-concept NIPD studies have been carried out using dPCR technologies to determine fetal *RHD *status in RhD-negative women^[55,58]^. Paternal exclusion testing by droplet digital PCR (ddPCR) is now also in routine clinical use in France for neurofibromatosis type 1 (*NF1*) and 2 (*NF2*), cystic fibrosis (*CFTR*), as well as several other monogenic conditions where pregnancies are at an increased risk (25% or 50%) of paternal inheritance, in cases with abnormal ultrasound findings, or a previously affected pregnancy^[42,59]^. Interestingly, Orhant *et al*. have assessed the use of ddPCR alongside a minisequencing protocol to detect and confirm fetal inheritance of a *de novo* missense variant in *FGFR3*, causative of achondroplasia^[60]^. As with RT-qPCR, clinical applications of dPCR to date are focused towards paternal and *de novo *inheritance patterns owing to the challenges associated with detection of maternally inherited variants.

### PCR-next-generation sequencing

PCR-NGS for NIPD involves targeted amplification of the genomic region containing the variant of interest using PCR, followed by NGS (otherwise known as massively parallel sequencing) to a depth of > 10,000X for visualisation and counting of the alleles present in the original cfDNA sample pool [Figure 1E]. The FF is simultaneously determined using a PCR-NGS single nucleotide polymorphism (SNP) panel, which typically contains heterogeneous SNPs along with a Y chromosome marker. PCR-NGS has been employed routinely through the UK National Health Service (NHS) to screen for a panel of *FGFR3* and *FGFR2 *recurrent pathogenic variants in fetuses with scan anomalies indicative of the associated conditions^[30,31,33]^, or for exclusion of a paternally inherited allele where a father is affected. Such tests, however, are suitable only for a small number of patients and therefore the use of this methodology was expanded to allow for bespoke testing to account for the extensive heterogeneity of pathogenic mutations causative of hereditary monogenic conditions^[61]^. Bespoke testing is now available for families at risk of autosomal recessive monogenic conditions where parents carry different pathogenic variants (i.e., paternal exclusion testing), or for paternally inherited autosomal dominant conditions. This testing can also be accessed to exclude a recurrence in couples with a previously affected pregnancy with a *de novo *pathogenic variant.^[32,41,61]^. The significant cost and time associated with case-by-case PCR-NGS test development have limited the clinical uptake of this technology; however, several improvements are being explored towards additional approaches with a broader target scope. Notably, the Danish service for fetal ABO blood group prediction also makes use of a simple PCR-NGS technology for gestational ages > 12 weeks^[27]^.

### Capture-based NGS

A commercially available NIPT for low-risk pregnancies marketed under the brand name Vistara (Natera, Inc., San Carlos, CA, USA) offers screening for 25 different conditions with autosomal dominant or X-linked inheritance patterns, with coverage across 30 genes and at a minimum of 9 weeks gestation^[39,62]^. The Vistara test employs NGS following probe-based capture to target several genes of interest [Figure 1F]. During library preparation, the DNA strands are labelled using unique molecular indexes (UMI) to facilitate filtering of sequencing and PCR artefacts from the data, allowing for accurate calling of low-level fetal-specific variants at a reduced depth than is possible with conventional PCR-NGS. Importantly, all positive results obtained using this method require confirmatory invasive testing. For this reason, Vistara is still a screening test and should not be considered diagnostic. While no false-positive or -negative results have been reported for this test, postnatal follow-up for result confirmation was obtained for only 53.6% of the pregnancies where Vistara was used^[62]^. Due to the low follow-up rate, the American Society for Obstetricians and Gynaecologists has released a statement recommending against the use of this test for low-risk screening in pregnancy owing to the lack of positive and negative predictive values that can be obtained^[63]^. Another limitation of this probe capture-based NGS approach is the incomplete coverage obtained across the targeted genomic region, leaving a variable but significant chance of missing pathogenic variants that exist within the gaps in sequencing. Although the average detection rate across all genes is ≥ 97%^[39,62]^, for some genes on the panel, it is as low as 47%^[64]^. Additionally, the fetal carrier status with respect to maternal pathogenic variants cannot be determined using this test^[62]^.

### Whole exome sequencing

Whole exome sequencing (WES) of maternal, paternal and fetal DNA obtained using invasive sampling methods (i.e., trios) has been shown to provide significant diagnostic uplift for rare monogenic conditions^[65]^. WES utilising cfDNA is an attractive target to screen for single gene disorders in both fetuses with anomalies detected by imaging and also in place of newborn screening programs^[66]^. In addition, it may provide a solution where invasive sample acquisition is not possible, for example, owing to a particular position of the placenta^[67]^. There have been a few studies on this, with Provenzano *et al*. successfully identifying autosomal recessive pathogenic variants in *FRAS1*^[67]^, and a *de novo *pathogenic variant in X-linked *STAG2*^[68]^. Another small study conducted on three pregnancies with structural anomalies detected by imaging, however, identified several technical challenges associated with trio WES on cfDNA^[66]^. As such, the authors recommend a more targeted approach^[66]^. Even so, non-invasive WES remains an attractive avenue for expanding the scope of NIPD in the future.

## TECHNIQUES USED TO DETECT MATERNALLY INHERITED VARIANTS

**Figure 2:**
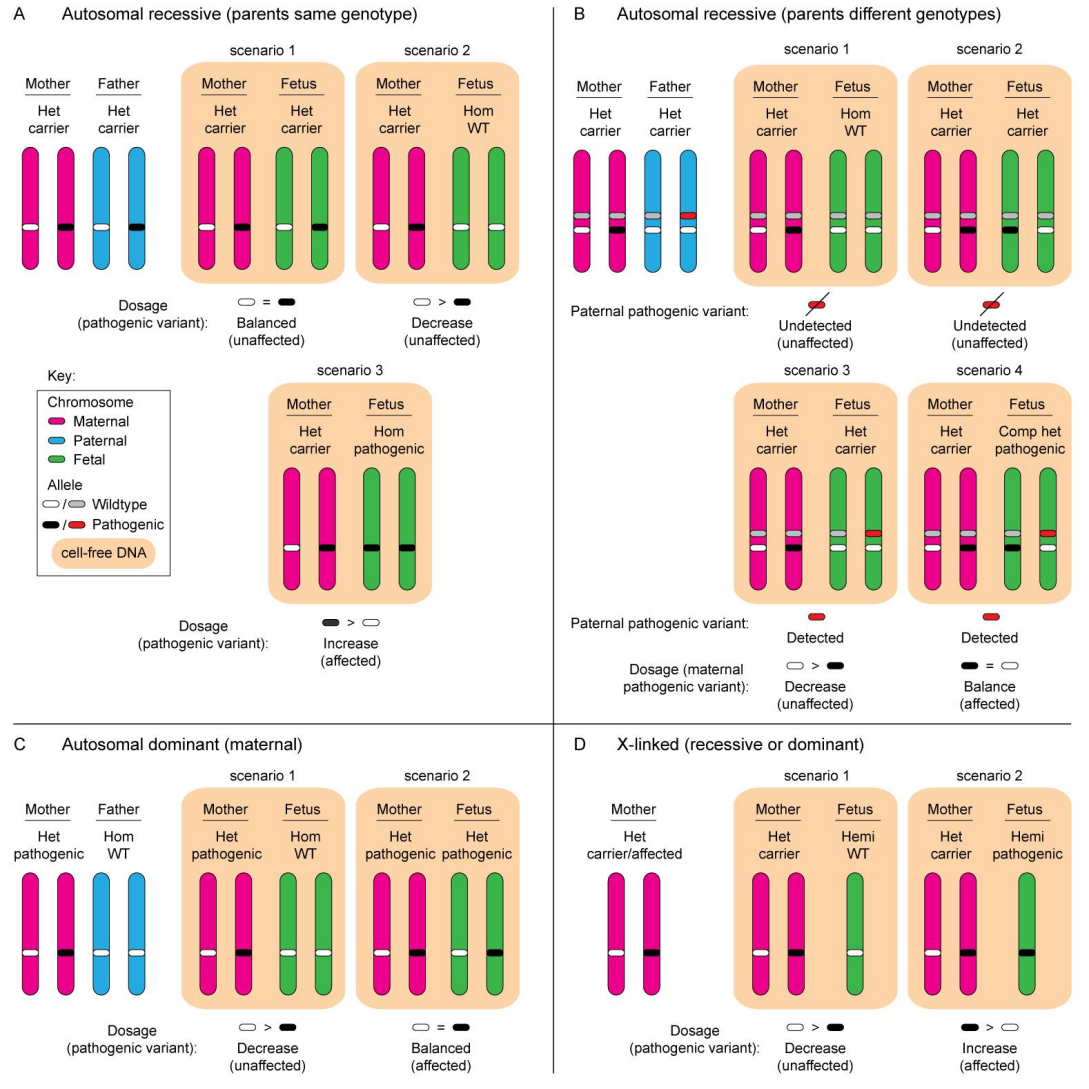
Relative mutation dosage (RMD) analysis for NIPD of maternally inherited pathogenic variants. A: Schematic depicting the relative mutation dosage (RMD) approach taken for NIPD of autosomal recessive conditions where the parents are both heterozygous carriers (het) for the same pathogenic variant at the same genetic locus. A balance or decrease in the quantity of the pathogenic variant (relative to the wildtype (WT) variant) indicates that the fetus is unaffected (scenarios one and two). An increase in the pathogenic variant indicates that the fetus is affected (scenario three). B: Schematic depicting the RMD approach taken for NIPD of autosomal recessive conditions where the parents are het for different pathogenic variants at different loci within the gene of interest. Testing for the paternal pathogenic variant can be carried out prior to RMD, whereby if it is not present (undetected), the fetus is unaffected (scenarios one and two). If detected, RMD can be performed. A decrease in the maternal pathogenic variant indicates that the fetus is unaffected (scenario three), while a balance indicates that the fetus is a compound het (comp het), and thus affected (scenario four). A similar approach can be taken for parents carrying different pathogenic variants at the same genetic locus (not shown), where dosage analysis of the maternal variant can be carried out if the paternal pathogenic variant is detected (similar to scenario three in A). C: For autosomal dominant conditions where the mother is het affected and the father homozygous (hom) unaffected, a decrease in the maternal pathogenic variant indicates that the fetus is hom unaffected (scenario one), while a balance between the pathogenic and WT allele indicates that the fetus is het affected (scenario two). D: For X-linked recessive or dominant conditions where the mother is het (carrier or affected, respectively), a decrease in the maternal pathogenic variant indicates a hemizygous (hemi) unaffected fetus (scenario one), while an increase indicates that the fetus will be hemi affected (scenario two). Confirmation of the fetal fraction is necessary to confirm a negative result where there is a balance in WT and pathogenic variants detected (A-C), or where the paternal pathogenic variant is not detected (B).

**Figure 3:**
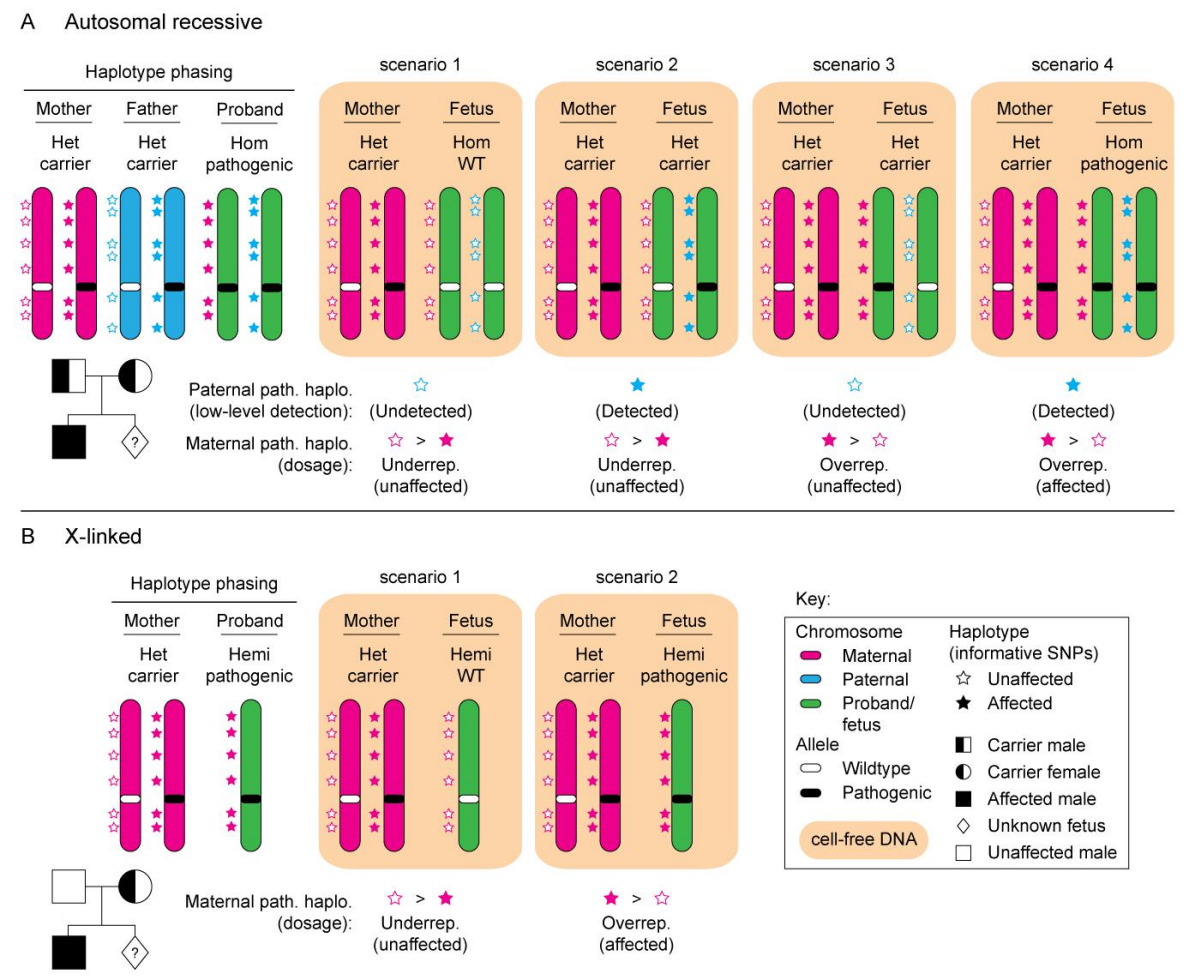
Relative haplotype dosage analysis (RHDO) for NIPD of maternally inherited pathogenic variants. A: Schematic depicting the relative haplotype dosage analysis (RHDO) approach for NIPD of autosomal recessive conditions where both parents are heterozygous carriers (het) for the same condition. Haplotype phasing is carried out on gDNA obtained from the mother, father, and proband to assign informative single nucleotide polymorphisms (SNPs) to a high- or low-risk allele. Informative SNPs are present at loci where the genotype differs between the mother and father. For simplicity, the proband is represented here as affected, but an unaffected proband can be used instead if necessary. RHDO is carried out on the cell-free DNA (cfDNA, orange highlighted area) by probe-based capture of the target region followed by short-read next-generation sequencing (NGS), SNP typing, fetal fraction (FF) calculation, and statistical analysis. The fetal genotype is determined to be unaffected where the paternal high-risk allele is not detected, or if there is low-level detection of the paternal high-risk allele but there is an underrepresentation of informative SNPs associated with the maternal high-risk allele (scenarios one, two and three). Conversely, an affected fetus is identified where the paternal high-risk allele is detected, and there is an overrepresentation of the SNPs associated with the maternal high-risk allele (scenario four). B: Schematic depicting RHDO for NIPD of male fetuses where the mother is het for an X-linked condition. Only maternal and male proband samples are required for haplotype phasing. The fetal genotype is determined to be unaffected (scenario one) or affected (scenario two) where the informative SNPs associated with the high-risk allele are under- or overrepresented in the cfDNA, respectively.

The high abundance of maternal cfDNA in circulation, and variation in FF between individual pregnancies, make detection of maternally inherited variants for NIPD particularly challenging. A small deviation from the expected threshold with respect to the FF must be detectable to ascertain whether the fetus has inherited the maternal pathogenic variant or not. Two main dosage-based methods have been developed to achieve this, namely relative mutation dosage (RMD) [Figure 2] and relative haplotype dosage analysis (RHDO) [Figure 3]. The primary difference between these two molecular counting approaches is that RMD involves the specific detection of a variant of interest, while RHDO involves the prior construction of the possible maternal and paternal haplotypes across the region, which are then used to infer inheritance of the low- or high-risk allele by the fetus. Additionally, RMD only requires a maternal blood sample, while conventional RHDO relies on both parental samples as well as an affected/unaffected proband.

### Molecular counting approaches

RMD measures the subtle differences between the expected and empirically measured levels of the variant of interest in cfDNA, provided that such differences exceed the inherent artefacts of the assay [Figure 2]. dPCR and barcode enabled NGS are the two major methods under development for RMD-based NIPD of maternally inherited variants.

#### dPCR-based relative mutation dosage

Proof-of-concept RMD-based NIPD studies have been carried out using dPCR technologies for several monogenic conditions^[69,70]^, including β-thalassaemia^[71,72]^, sickle cell disease^[73,74]^, monogenic diabetes^[75]^, haemophilia^[76,77]^, cystic fibrosis^[59,78]^, methylmalonic acidaemia^[79]^, inherited deafness^[80,81]^, as well as a small bespoke cohort with a number of different inheritance patterns^[73]^. A ddPCR-based RMD method has also been employed for NIPD of families at risk of spinal muscular atrophy, involving molecular counting of the copies of the *SMN1* gene in cfDNA of mothers who carry only one functional copy^[82]^.

A number of statistical analysis methods have been applied to fetal genotype prediction following dPCR for RMD of maternally inherited variants. For instance, the sequential probability ratio test (SPRT), which is also the method commonly used with RHDO, has been the analytical method of choice in several studies^[69,71,74,76,77,83]^. Z-score, also employed in the context of RMD using ddPCR towards NIPT for fetal aneuploidy^[84]^, has also been selected for analysis^[70,72,79,85]^, as has a chi-squared test^[81]^, and a custom Markov chain Monte-Carlo (MCMC) Bayesian approach using JAGS (just another Gibbs sampler)^[75]^.

Of concern is the small but significant number of misclassified and inconclusive results which have been reported in several studies that have applied dPCR to detect maternally inherited variants in cfDNA for NIPD of monogenic conditions^[70-72,74,75,83,85]^. The unknown cause of such incorrect results and the wide range of different statistical approaches which have been applied to the various published studies is somewhat problematic. Comparative research that assesses the performance (i.e., sensitivity and specificity) of the different statistical tests could provide valuable insights towards the potential clinical utility of dPCR for NIPD. A recent publication compared the SPRT, Z-score and Markov chain Monte-Carlo (MCMC) Bayesian approach using JAGS analysis methods on a cohort of 124 cases, demonstrating that they all performed similarly^[73]^; however, two of the cases were still incorrectly classified by all three methods. While additional statistical methods are useful for re-analysing inconclusive results, this does not overcome the important challenge of misclassification by dPCR-based RMD.

Several other disadvantages of RMD using dPCR are the narrow mutation size range that can be detected, prior knowledge of the variant of interest, and the requirement for sufficient starting genomic material as well as a minimal FF. The assay time for dPCR requires only 2-3 days of lab work, but the design, ordering and optimisation of bespoke targets can take several weeks, which is potentially an unacceptable length of time for prenatal testing^[69]^. As such, other strategies that can be performed with only a maternal sample, but leverage the broader target range and sensitivity of PCR-NGS, are also in the developmental pipeline to overcome these challenges.

#### NGS-based relative mutation dosage

One of the limitations of using NGS for molecular counting in the context of maternally inherited variants is the inability to quantify the number of input DNA molecules, particularly with the inclusion of a PCR enrichment step. A modified NGS-based RMD approach, with no requirement for paternal or proband samples, has recently been described for the detection of maternally inherited pathogenic variants in the *HBB* gene, causative of sickle cell disease^[86]^. Specifically, UMIs were introduced into individual cfDNA fragments through targeted PCR, followed by massively parallel sequencing. Prior to this, cfDNA size selection was carried out to enrich for shorter cfDNA typically considered to be of fetal origin^[86]^. Unpredictable loss of DNA during library preparation, as well as errors and biases introduced during the PCR step, are overcome as sequencing reads containing the same UMI are counted only once. The number of unique DNA molecules and the FF can thus be calculated more accurately, and the sensitivity and specificity when using this UMI NGS-based RMD approach were reported to be 85.7% and 97.6%, respectively^[86]^. A similar approach, coined cfDNA barcode‐enabled single‐molecule test (cfBEST), was taken as a proof-of-concept study for the detection of maternally inherited variants in *HBB*^[87]^ and *DMD*^[88]^, with a sensitivity and specificity of > 99%^[87,88]^.

A modified UMI-based NGS approach, validated originally using Sanger sequencing for variants causative of Wilson disease (*ATP7B*)^[89]^, has also been developed for NIPD of various monogenic conditions with pathogenic variants causative of autosomal recessive non-syndromic hearing loss (*GJB2, GJB3, SLC26A4*, *RNR1*, *TRNL1*, and *COX1*)^[90,91]^, β-thalassaemia (*HBB*)^[92,93]^, phenylketonuria (*PAH*)^[94,95]^, and methylmalonic acidaemia cb1C type (*MMACHC*)^[96]^. This methodology was called circulating single-molecule amplification and resequencing technology (cSMART). cSMART involves a preamplification reaction that enables tagging of individual target molecules with UMIs, as well as enrichment of low frequency target molecules. The tagged DNA is then circularised, and inverse PCR carried out prior to paired-end NGS, whereafter uniquely barcoded molecules are counted only once^[89]^. A panel of 76 SNPs with high heterozygosity in the population^[97]^ was used in several of these studies for FF quantification^[90,93,95,96]^. A multi-amplicon PCR-NGS approach, incorporating both PCR and sequencing error correction strategies, with FF calculation by methylation-specific restriction enzyme digestion followed by qPCR, has also been demonstrated for NIPD of sickle cell disease (*HBB*)^[98]^. This approach was reported to have a sensitivity and specificity of 94% and 88%, respectively, where false positives were obtained at a FF < 4% and false negatives at < 1%^[98]^.

In the commercial sector, the UNITY Screen™ (BillionToOne Inc., Menlo Park, CA, USA) is an NGS-based test that does not require a paternal sample and is available for low-risk pregnancies from 10 weeks gestation. The UNITY Screen™ is a maternal carrier test covering four autosomal recessive conditions, namely cystic fibrosis, spinal muscular atrophy, and α- and β-haemoglobinopathy, with reflex NIPT of the cfDNA for the paternal allele where the mother is determined to be a carrier^[38,99]^. If the paternal allele is detected in the cfDNA, inheritance of the maternal allele is ascertained using an RMD quantitative counting template (QCT) approach^[38]^. A performance evaluation was carried out on 9,151 pregnant individuals who had undertaken the UNITY Screen™, with a follow-up rate of 10.3% at the time of reporting and demonstrating 98.7% sensitivity, 99.4% negative predictive value, and 48.3% positive predictive value^[100]^. Another evaluation carried out over a three-year period compared the UNITY Screen™ to conventional carrier testing which requires testing of a paternal sample where the mother is determined to be a carrier^[99]^. Results from this assessment demonstrated that the UNITY Screen™ is more than twice as sensitive (98.5% relative to 41.5%, respectively), and that there was a 62% reduction in cost associated with identifying an affected pregnancy. The major benefit of the UNITY Screen™ is that logistical barriers to obtaining the paternal sample, as is required for conventional carrier screening, are avoided.

While the UNITY Screen™ provides a fetal risk score, confirmation of a result deemed to be above a risk threshold is still required by performing an invasive follow-up test in an accredited clinical facility^[99]^. Furthermore, the panel used for the screen does not cover the entirety of each of the target genes responsible for cystic fibrosis (*CFTR*) or haemoglobinopathies (*HBB*). Even so, such tests expand non-invasive testing for monogenic conditions to low-risk maternal groups and reduce the number of women undergoing invasive testing. Crucially, adequate counselling and a high postnatal result follow-up rate are required to ensure that patients are fully aware of the meaning of their results, and that the community has confidence in the sensitivity and specificity of the results that are reported. Importantly, a greater effort towards clinical validation is required as a 10% follow-up rate is inadequate for clinical service.

#### Relative haplotype dosage analysis

In the clinical setting, RHDO is employed for NIPD of maternally inherited variants in high-risk pregnancies [Figure 3]. In 2010, Lo *et al*. developed RHDO for NIPD of maternally inherited variants causative of β-thalassemia^[4]^. RHDO does not directly measure the pathogenic variant, but genotyping is instead inferred through haplotyping of the high- and low-risk alleles constructed from genomic DNA (gDNA) obtained from both parents, as well as an affected or unaffected family member (i.e., a proband, usually a sibling) [Figure 3A]. Of note, RHDO for X-linked inheritance patterns does not require a paternal sample [Figure 3B]. Typically, RHDO is carried out using capture-based target enrichment, followed by NGS and statistical analysis for haplotype phasing and genotyping^[4]^. The statistical method most widely applied to RHDO analysis is SPRT^[4,34,36,37,44,61,77,101,102]^, although other Bayesian approaches have also been reported in the literature (e.g., the hidden Markov model)^[103,104]^. This review will focus primarily on the SPRT approach.

Over the past decade, RHDO has been introduced into routine clinical use for NIPD of maternally inherited pathogenic variants for cystic fibrosis (*CFTR*)^[36]^, spinal muscular atrophy (*SMN1*/*SMN2*)^[37]^, and congenital adrenal hyperplasia (*CYP21A2*)^[105]^, as well as for X-linked Duchenne and Becker muscular dystrophies (*DMD*)^[34]^. RHDO is also under development for several other autosomal recessive conditions^[101,106]^, and for maternal inheritance of autosomal dominant retinoblastoma (*RB1*)^[102]^. The high sensitivity achieved with RHDO has resulted in its clinical implementation, and a major advantage is that this approach can be used even when parents carry the same pathogenic variant. However, the requirement for samples from both the father as well as an appropriate affected or unaffected family member/proband, along with the maternal blood sample, restricts the scope of families eligible for RHDO and introduces logistical barriers with regard to sample acquisition. Another limitation of RHDO is that a recombination event at or near the variant genomic locus may cause haplotyping to fail, or the reporting of incorrect results, although cross-over events have reportedly been correctly identified using RHDO^[36]^. Furthermore, RHDO is not currently offered to consanguineous couples owing to the increased risk of a shared haplotype and low numbers of informative SNPs.

SNP genotypes that differ between the maternal and paternal samples are referred to as informative SNPs, as these inform the construction of the fetal haplotype blocks. To assign identity to such SNPs, the raw sequencing data must pass through a bioinformatics pipeline which is subdivided into the primary, secondary, and tertiary analysis phases^[107]^. The primary analysis excludes sequencing reads which do not pass a predetermined quality score threshold. In the second phase, sequence alignment against the reference human genome is carried out, at which point reads containing repetitive sequences or high homology to multiple regions in the genome may be excluded, resulting in gaps or low sequencing coverage at these loci. Even so, the RHDO methodology is generally not negatively impacted by sequence coverage gaps owing to the large genomic regions (up to several Mb of DNA) across which informative SNPs are assessed. The final step in the pipeline involves variant calling with the purpose of identifying all SNVs within the mapped reads. As part of the tertiary analysis, further useful information is included, such as in which gene and feature the variant is located and the functional impact of the variation (missense, nonsense, synonymous, stop-loss, and so on).

From the processed NGS data, RHDO is performed by haplotype phasing, SNP typing and subtyping, SNP counting, FF calculation, and statistical analysis. In the case of X-linked conditions for NIPD of male fetuses, paternal genotype information is not required for RHDO and a male reference sample is sufficient to provide the haplotype linked with either the pathogenic (if an affected male proband is used) or normal (if an unaffected maternal grandfather proband is used) copy of the gene of interest^[34,35]^. Haplotype blocks are created using statistical methods to infer inheritance of the high- or low-risk allele. In the case of autosomal recessive conditions where parents carry different pathogenic variants, if the paternal high-risk allele is determined to have been inherited by the fetus, analysis is continued to determine inheritance of the maternal allele. For example, when using SPRT analysis, fetal haplotype blocks are constructed based on the proportion of each SNP present in the cfDNA (relative to the FF), progressing from both ends of the target region. For statistical analysis, the FF is used to obtain lower and upper boundary classification thresholds against which a cumulative score of each SNP is compared. Importantly, the mode of inheritance is also taken into consideration for these calculations. Statistical analysis is carried out until significance is reached, and a fetal haplotype block can be assigned to either the high- or low-risk maternal allele. The overall haplotype construction then informs maternal inheritance and can indicate whether a recombination event has occurred within the target region or not. Crucially, it can be difficult to ascertain if such a recombination event occurred in the proband or the fetus, and other methods that do not rely on a proband sample (i.e., proband-free RHDO) are being developed to simplify the RHDO methodology. Proband-free RHDO has the further advantages of streamlining the pre-processing requirements for sample acquisition and opening up RHDO to families where a proband sample is unavailable.

#### Proband-free RHDO with short-read sequencing

**Figure 4:**
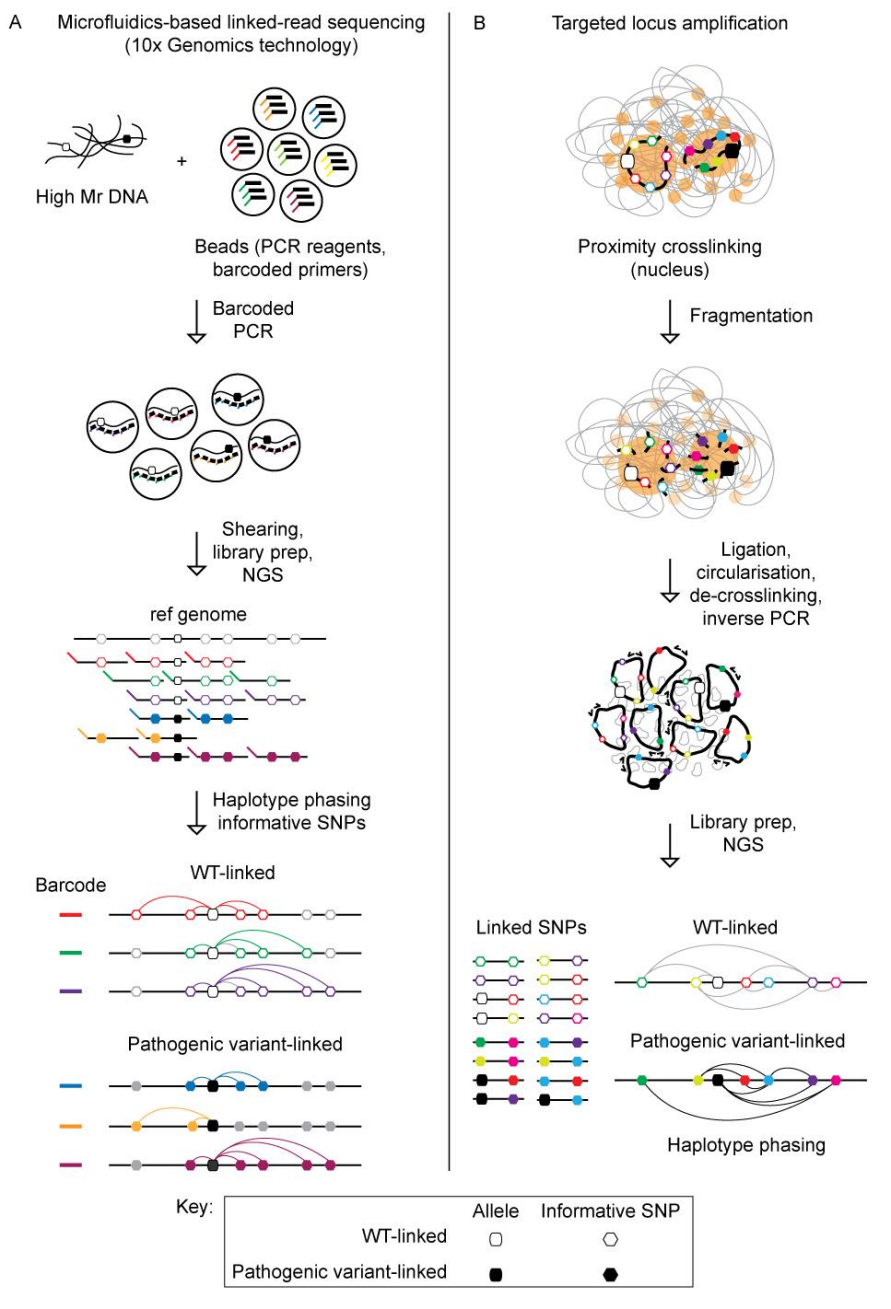
NIPD technologies applicable to proband-free RHDO haplotyping phasing. A: Schematic depicting the workflow carried out for microfluidics-based linked-read sequencing (using the 10x Genomics technology) which has been employed for proband-free RHDO. High molecular weight (Mr) DNA is combined with beads containing uniquely barcoded primers as well as all required polymerase chain reaction (PCR) reagents. Several PCR reactions are carried out along the length of the high Mr DNA fragment, introducing a unique barcode into each PCR product. The DNA is sheared, and short-read next-generation sequencing (NGS) carried out. Reads are aligned to the reference human genome and PCR duplicates are excluded from the analysis. Haplotype phasing is carried out by assigning informative single nucleotide polymorphisms (SNPs) and the variant of interest to haplotype blocks of high- or low-risk based on the premise that reads with the same barcode originated from the same fragment of high Mr DNA. RHDO is then carried out on the cell-free DNA (cfDNA) as previously described [Figure 3]. B: Schematic depicting the workflow carried out for targeted locus amplification (TLA), which has been employed for proband-free RHDO. Nuclear genetic material in close proximity, typically intrachromosomal, is crosslinked and then fragmented. DNA fragments are re-ligated in a random manner and circularised such that informative SNPs from closely associated genomic regions are physically linked. De-crosslinking and inverse PCR are carried out in preparation for either short- or long-read NGS. Reads with > 1 informative SNPs are retained, and SNP linkage analysis is performed to generate haplotype blocks used for haplotype phasing of the high- and low-risk alleles.

Proband-free RHDO is a direct haplotyping approach that has recently emerged as an innovative solution for addressing the challenges currently faced with conventional RHDO. Microfluidics-based linked-read sequencing is one such approach, making use of the 10x Genomics (CA, USA) Chromium™ library barcoding technology to achieve haplotype phasing^[106,108-111] ^[Figure 4A]. Briefly, high molecular weight maternal and paternal gDNA fragments are separated into individual bead-based reaction chambers wherein PCR using unique 10x barcoded primers is carried out at regular intervals across the target region^[106]^. Shearing of the DNA followed by short-read whole genome sequencing (WGS) enables the identification of informative SNPs, which can be linked to either the pathogenic or WT variant of interest based on shared barcode sequences. SPRT is then carried out on the cfDNA as previously described for the RHDO technology. Proof-of-principle studies of this approach have demonstrated clinical utility for fetuses of families at risk for autosomal recessive conditions including congenital adrenal hyperplasia (*CYP21A2*), β-thalassaemia *(HBB*), and Ellis-van Creveld syndrome (*EVC*, *EVC2*)^[106]^, as well as the X-linked conditions haemophilia (*F8*, *F9*), Hunter syndrome (*IDS*, *IDS2*)^[106]^, and Duchenne or Becker muscular dystrophy (*DMD*)^[108]^. Notably, for X-linked conditions, direct haplotyping based on the 10x Genomics technology requires only a maternal gDNA sample, unlike for conventional RHDO, which necessitates the inclusion of a proband sample^[106,108]^. An additional advantage of the linked-read strategy is that a recombination event can be directly attributed to the fetus, while for RHDO, it is challenging to determine if this phenomenon is instead present in the proband sample^[108]^. The direct haplotyping approach has also been demonstrated in the context of autosomal dominant triplet-repeat expansion conditions, such as myotonic dystrophy type 1 (*DMPK*)^[109,111]^ and Huntington’s disease (*HTT*)^[111]^. Detection of such expansions is difficult to achieve using other molecular counting methodologies, particularly where the overall length of the repeat expansion exceeds the lower limit of detection for short-read sequencing.

The clinical utility of the microfluidics-based linked-read approach is limited as the reliance on WGS is expensive and the 10x Genomics technology is no longer commercially available for cfDNA testing. Another proband-free RHDO approach that has been developed to overcome these barriers is targeted locus amplification (TLA)^[112,113] ^[Figure 4B]. The premise of TLA is that genomic DNA in close spatial proximity within the nucleus is likely to be intrachromosomal, and so crosslinking can physically tether informative SNPs of a particular haplotype to one another^[112,114]^. Briefly, haplotyping by TLA involves gDNA crosslinking, fragmentation, circularisation, and inverse PCR followed by targeted short-read sequencing using either Sanger or NGS technologies^[110,112,113]^. Reads that contain > 1 informative SNP are selected for haplotype phasing. This technology was first reported in the context of NIPD for several autosomal recessive conditions, including cystic fibrosis (*CFTR*), β-thalassaemia (*HBB*), and congenital adrenal hyperplasia (*CYP21A2*)^[113]^. More recently, TLA has also been applied to methylmalonic acidaemia (*MMACHC*), α- and β-thalassaemia (*HBA*, *HBB*), phenylketonuria (*PAH*), polycystic kidney disease (*PKHD1*), and autosomal recessive non-syndromic hearing loss (*GJB2*)^[110]^. TLA also has the potential to detect large structural variants and chromosomal rearrangements^[115]^.

### Long-read sequencing

The recent discovery of long cfDNA fragments in maternal circulation has provided an opportunity to carry out long-read sequencing directly on cfDNA, further increasing the range of conditions that can be tested or allowing for the development of novel NIPD techniques that can improve currently available clinical tests^[15]^. However, no studies have yet reported the use of long-read sequencing on cfDNA for NIPD. The technology has instead primarily been applied to haplotype phasing of parental gDNA. For example, in the context of proband-free RHDO, long-read sequencing has allowed for the detection of target linked SNPs, thereby reducing the size of the haplotyped region and the number of haplotype blocks required to reach statistical significance^[116]^.

Another method that leverages long-read sequencing technology, but does not rely on haplotype construction, has also been explored in the research setting with a particular focus on spinal muscular atrophy^[117]^. Chen *et al*. describe the application of PacBio long-read sequencing in order to determine the phase and copy number of the highly homologous *survival of motor neuron 1* (*SMN1*) and *SMN2 *genetic loci^[117]^. These genes both have variable copy numbers across the population and correlate negatively with the severity of spinal muscular atrophy. Although Chen *et al*. do not make direct mention NIPD, the accurate and simultaneous determination of *SMN1* and *SMN2 *copy number and phase has significant potential to be developed into a useful screen for silent carriers in the population (i.e., those with monoallelic multi-copy *SMN1*). Furthermore, this approach could be applied to parental haplotyping and proband-free RHDO in the future.

Another area of interest with respect to long-read sequencing for NIPD is tissue-specific epigenetic profiling. Traditionally, bisulphite sequencing (BS-seq) is carried out to detect methylated gDNA; however, this technique is highly damaging and results in DNA degradation^[118]^ which would counteract the potential benefits offered by long-read sequencing. A recent study was conducted using PacBio sequencing, leveraging the differences in DNA polymerase kinetics to identify base modifications such as methylation^[119]^. These differences can be interpreted by analysing the change in inter-pulse duration (i.e., the time between two fluorescence pulses) and pulse width (i.e., the length of time of one fluorescence pulse). A convolutional neural network was trained using data generated from this sequencing methodology in order to assign methylation status, with results comparable to traditional BS-seq data. Using this model, long plasma DNA molecules were sequenced and assigned a methylation pattern which was then compared to reference tissue methylomes from BS-seq data. The authors have demonstrated that from these patterns, it is possible to identify the tissue-of-origin of each plasma DNA molecule, specifically cfDNA with a buffy coat methylation pattern which is determined to be maternally derived, or a placental methylation pattern which is determined to be fetally derived. Shallow WGS using Oxford Nanopore Technologies (ONT, Oxford, UK) long-read sequencing has been shown to be useful for detecting cell-type and cancer-specific methylation changes and cancer-associated fragmentation patterns in liquid biopsies^[120]^. In the context of NIPD, the ability to assign individual cfDNA molecules to a specific tissue may enable the direct interrogation of which allele a fetus has inherited, even for maternally inherited variants, without the need for dosage-based analytical approaches such as RMD or RHDO. Furthermore, this approach may be useful for FF calculation in a manner that does not require prior knowledge of informative paternal SNPs and can be applied independently of fetal sex. When considering the utility of epigenetic biomarkers for NIPD, a crucial research avenue to be explored is the impact that an affected pregnancy may have on the cfDNA methylome, both at a global scale as well as at the region of interest, and whether this can be predicted or not, so as to avoid potential confounding factors.

## THE IMPORTANCE OF FETAL FRACTION QUANTIFICATION

Accurate quantification of the abundance of cffDNA in the analysed cfDNA sample is paramount to ensuring quality control and statistical confidence for NIPD of monogenic conditions. Several biological factors are known to cause FF variability, although the culminating effect of these is, to a large extent, unpredictable and so are not currently considered in clinical workflows^[121]^. FF estimation is, therefore, primarily a function of the methodology and bioinformatic algorithms employed. Lun *et al*. demonstrated the importance of assay sensitivity, reporting that a higher FF than expected was detected when using microfluidics-based dPCR compared to conventional RT-qPCR^[122]^. NIPT for fetal aneuploidy detection typically applies a FF cut-off of > 4%^[13,123,124]^, and confirmation of the FF is essential for the interpretation of negative results as these could be due to an absence or insufficiency of cffDNA in the sample. Similarly, for NIPD in the context of paternally inherited and *de novo *variants, it is important to check the depth of the assay and confirm the presence of fetal DNA in the sample. The FF threshold required for accurate NIPD in the context of maternally inherited variants is typically > 4% in order to achieve statistical significance^[80,98]^. 

The most straightforward approach to FF quantification involves detection of circulating Y chromosome genetic material, which has been done using RT-qPCR^[22,23,125]^, dPCR^[45,55,80]^, and NGS^[126,127]^, but is necessarily limited to male pregnancies. Molecular counting of paternally inherited SNPs offers a fetal sex-independent approach to FF quantification and has been achieved using dPCR^[69]^ and NGS^[14,39,102,127-129]^. Differential methylation-based approaches have also been undertaken^[42,59,60,83,98,130-133]^ as cffDNA is hypomethylated relative to maternal cfDNA, believed to be the result of active genome-wide transcription during fetal development^[132,134]^. Other approaches that involve machine learning include a SNP- and fetal sex-independent method for FF determination which interrogates overrepresentation of differentially methylated regions between placental (fetal) and haematopoietic (maternal) derived cfDNA^[135]^. Another machine learning approach based on the phenomenon that cffDNA is, on average, shorter than maternal cfDNA, involved paired-end short-read sequencing and size profiling for FF quantification (SeqFF tool)^[127]^. A finding from this study is that the cffDNA is non-uniformly distributed across the genome^[127]^; however, constant distribution of cfDNA fragments has been observed by Lo *et al*.^[4]^. Further research into this phenomenon is required for confidence in FF quantification strategies that rely on paternally inherited SNPs present at distant loci relative to the variant of interest. Other studies have employed cfDNA size-based ^[136]^ and nucleosome track-based^[137]^ FF quantification methods.

## CELL-BASED NIPD

Cell-free fetal nucleic acids are accompanied by a low number of fetal cells in the maternal bloodstream, namely trophoblastic cells derived from the placental tissue and early erythrocytes (i.e., red blood cells), which are also specific to the ongoing pregnancy^[138,139]^. These populations of cells can theoretically be isolated from peripheral maternal blood from as early as 6 weeks of pregnancy, although reportedly more reliably from 10-14 weeks, and offer a source of intact fetal chromosomal DNA for NIPD of monogenic conditions^[140-143]^. Obtaining a pure fetal cell population provides the benefit of having intact gDNA within which more complex pathogenic variants can be detected^[144]^, such as triplet repeat expansions^[145]^. Furthermore, obtaining high molecular weight fetal gDNA negates the need for RHDO or RMD as maternal background DNA is not present in this sample type.

The field of single-cell omics has expanded rapidly in the past decade, and high sensitivity methods are now available, which could facilitate fetal genotyping on cell-derived fetal DNA obtained via non-invasive sampling methods^[143]^. Cell-based NIPD has been investigated in the research setting for cystic fibrosis^[143]^, congenital deafness and ichthyosis ^[146]^, and Huntington’s disease^[145]^, as well as for confirmation of an unaffected fetus following preimplantation genetic testing^[147]^. A major consideration of this strategy, however, is the way in which a pure population of fetal cells can efficiently and reliably be obtained from maternal circulation^[148]^. Extremely low yields have been reported in the literature thus far (~1.2-2.5 cells/mL of blood^[147,149]^), although an accurate estimation may be dependent on the method used for fetal cell enrichment. Further research is required to assess the true abundance of fetal cells in maternal circulation, as well as the feasibility of cell-based NIPD for monogenic conditions. Furthermore, the methods required to obtain pure populations of fetal cells are laborious and not every diagnostic laboratory facility will have access to the appropriate equipment and diagnostic setup. A semi-invasive method involving fetal cell retrieval and isolation from the cervix (TRIC) has the potential to offer an intermediary avenue towards obtaining a sufficient quantity of fetal-derived mononuclear extravillous trophoblasts (~500-1500 cells), a procedure that is technically comparable to that routinely performed for cervical cancer screening^[150,151]^. Cell-based NIPD has the potential to further revolutionise the field of prenatal genetic testing for monogenic conditions and research is ongoing in this field to circumvent the limitations currently hindering its clinical implementation.

## CONCLUSIONS

The discovery of cffDNA in maternal circulation has resulted in a paradigm shift for prenatal genetic testing towards non-invasive sampling methods. PCR- and NGS-based approaches allow for detection of paternally inherited and *de novo* variants in a relatively straightforward manner. The majority of ongoing research for NIPD of monogenic conditions is focused on overcoming the challenges surrounding accurate detection of maternally inherited pathogenic variants through the use of molecular counting approaches such as RMD and RHDO. A major logistical barrier to RHDO is the requirement for additional samples from family members to facilitate haplotype construction. As such, proband-free RHDO and direct haplotyping methods are currently being developed to simplify the existing workflow. The development of long-read NGS also has the potential to lend greater opportunity towards achieving this goal.

The list of diagnosed rare monogenic conditions for which the genetic cause has been identified is ever-growing. Even so, the vast number of pathogenic variants in existence, or which could arise, makes the development of an all-encompassing NIPD test unfeasible. While targeted case-by-case testing for rare monogenic conditions is achievable in many instances, strategies that reduce the number of bespoke tests required will be highly beneficial for reducing the cost and time associated with NIPD in the clinical setting. Important future work is required to determine the earliest timepoint, as well as the minimum FF threshold required, to achieve consistently accurate NIPD results for each assay used and mode of inheritance under investigation. dPCR and NGS offer exciting new avenues for NIPD scope expansion, although the relatively high cost of high-throughput sequencing approaches is still somewhat prohibitive in the clinical setting, and equity of access to such technologies, should they become available, is an ethical concern. Commercially available tests offer an expansion of access to low-risk pregnancies; however, stringent validation and follow-up studies, as well as a greater focus on pre- and post-test counselling, are essential to ensuring correct expectation and interpretation of results by service users. The field of NIPD for monogenic conditions is evolving rapidly, and exciting new technology development is on the horizon, which is likely to have a significant and positive impact on prenatal care in the near future.

## DECLARATIONS

### Copyright

© The Author(s) 2023.
